# Fractional exhaled nitric oxide (FeNO): the future of asthma care?

**DOI:** 10.3399/bjgp23X735813

**Published:** 2023-12-01

**Authors:** Kay Wang, Carol Stonham, Christine Rutherford, Ian D Pavord

**Affiliations:** Clinical Professor in Primary Medical Care, Primary Care Research Centre, School of Primary Care, Population Sciences and Medical Education, University of Southampton, Southampton.; Nurse Practitioner, NHS Gloucestershire Integrated Care Board.; Patient representative.; Professor of Respiratory Medicine, Respiratory Medicine Unit and Oxford Respiratory NIHR Biomedical Research Centre, Nuffield Department of Clinical Medicine, University of Oxford.

## BACKGROUND

Asthma affects around one in 30 people and contributes to nearly half a million deaths worldwide every year.^1^ It is estimated that asthma will cost the UK NHS £1.3 billion in 2023, including costs relating to primary care contacts and prescription of treatments for asthma.^2^

Treatments that aim to minimise risk of acute asthma exacerbations focus on reducing airway inflammation. However, despite significant advancements and increasing expenditure on asthma medications, incidences of asthma-related admissions to hospital and deaths have not improved over the last decade.^3^ This issue highlights the need for a more personalised ‘treatable traits’ approach^4^ whereby treatments are targeted and optimised based on an individual’s underlying disease mechanisms rather than diagnostic labels encompassing varying patterns of airway dysfunction and symptom expression, which may not correlate with levels of airway inflammation.^5^

**Figure 1.**
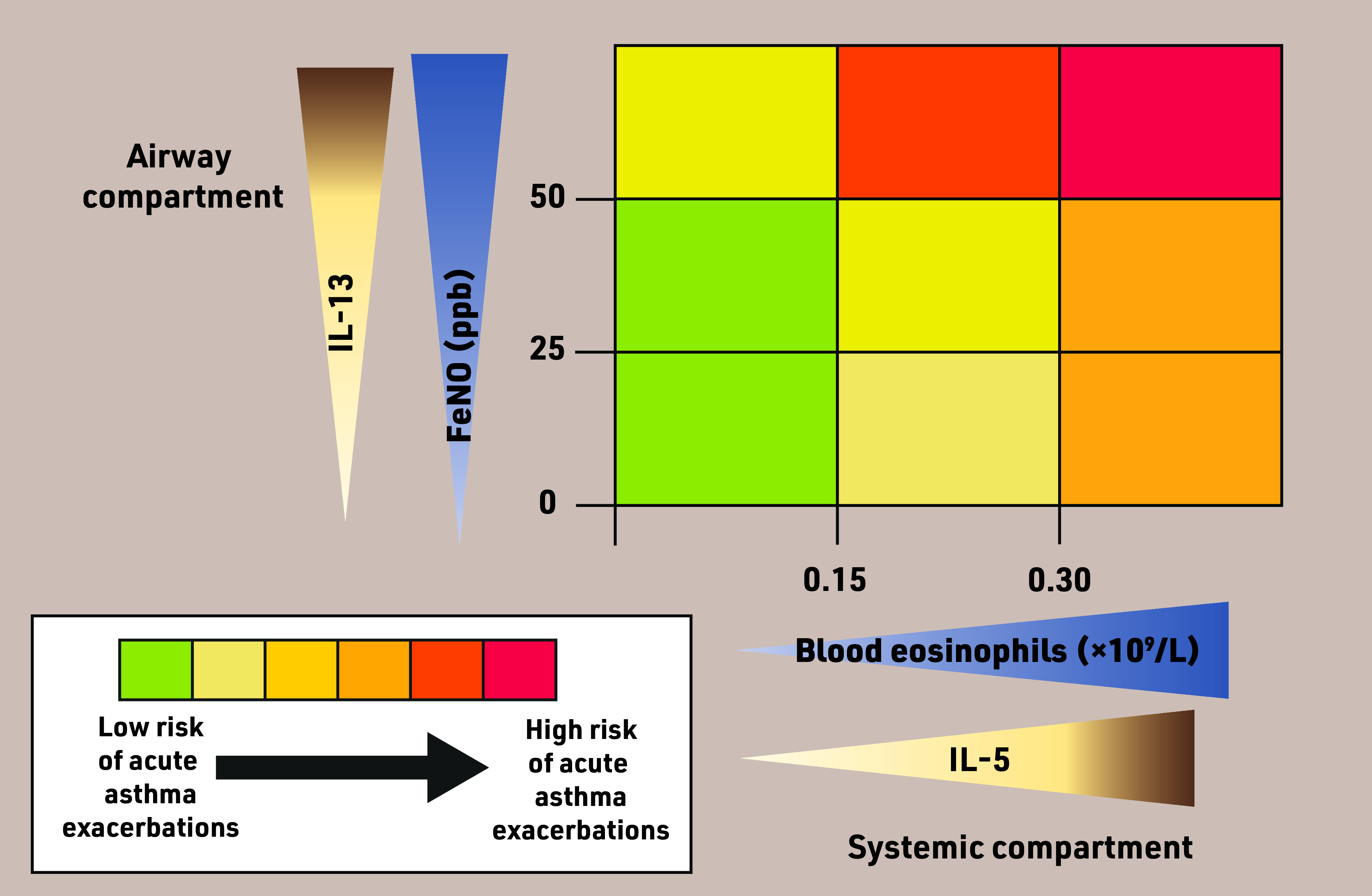
*FeNO and blood eosinophils in predicting risk of acute asthma exacerbations. High FeNO (50 ppb or higher) and high blood eosinophils (0.30 × 10^9^/L or higher) are associated with increased risk of acute asthma exacerbations. FeNO is driven by IL-13 and reflects the degree to which blood eosinophils are attracted to the airways. Blood eosinophils are driven by IL-5 and reflect the systemic pool of available eosinophils. High FeNO and blood eosinophils in combination are associated with the greatest risk of exacerbations. FeNO = fractional exhaled nitric oxide. IL-5 = interleukin-5. IL-13 = interleukin-13. ppb = parts per billion. This figure is based on data presented by Couillard* et al *in relation to the derivation of a prototype risk score for acute asthma exacerbations.^9^*

Fractional exhaled nitric oxide (FeNO) is a non-invasive breath test that provides an objective measure of type-2 airway inflammation, which is typically steroid responsive. Higher FeNO levels have been shown to predict increased risk of acute exacerbations in patients with moderate-to-severe asthma.^6^ Risk of exacerbations is observed to be even higher when both FeNO and blood eosinophils are raised.^6^^,^^7^ It is proposed that, while FeNO reflects the degree to which blood eosinophils are attracted to the airways, the blood eosinophil count reflects the systemic pool of available eosinophils.^8^ These two components interact synergistically to increase exacerbation risk (Figure 1).^9^

Stratified analyses of data from randomised controlled trials (RCTs) studying the effectiveness of inhaled corticosteroids (ICS) or biologic therapies in patients with mild, moderate, and moderate-to-severe asthma have shown these treatments to be more effective in patients with biomarkers indicating high levels of type-2 inflammation.^10^

## FENO-GUIDED ASTHMA MANAGEMENT: WHAT IS THE EVIDENCE?

The 2017 National Institute for Health and Care Excellence (NICE) guideline on asthma diagnosis, monitoring, and management^11^ recommended that FeNO should be used to support diagnosis of asthma. A high FeNO result is reported to have a positive predictive value of 54% to 95% in adults and 90% in school-aged children.^12^ However, patient-reported symptoms have poor sensitivity and specificity, and published data on peak flow monitoring are unreliable as it is unclear whether monitoring took place when patients were symptomatic.

Home peak flow and symptom monitoring, spirometry, and serial bronchial challenge testing in patients with physician-diagnosed asthma recruited from 10 cities across Canada found that around one-third of patients had been incorrectly diagnosed.^13^ However, more than half of patients with asthma may have normal spirometry^12^ and bronchial challenge testing is not readily accessible in primary care.

The 2017 NICE guideline^11^ also highlighted the need for more robust evidence to determine the role of FeNO in guiding management of asthma. A systematic review of RCTs comparing FeNO-guided asthma management strategies with other strategies (mainly symptom guided)^14^ found that the number of patients who experienced an acute exacerbation during follow-up periods lasting 18 to 52 weeks was significantly lower in patients whose management was guided by FeNO; numbers needed to benefit were 12 in adults and 9 in children. However, the implications of these findings for clinical practice are unclear because of the considerable heterogeneity between RCTs in relation to study population characteristics, algorithm decision points (including FeNO categorisation cut-off values), and definitions of acute exacerbations. These factors are highly relevant in determining the extent to which a FeNO-guided algorithm can provide sufficiently different management recommendations and clinical outcomes versus decisions made without FeNO.^15^

A recent RCT conducted in children with asthma who had experienced an acute exacerbation within the last 12 months^16^ found that adding FeNO to a symptom-guided management strategy did not lead to a significant reduction in acute exacerbations. However, baseline FeNO values of trial participants were typically consistent with low type-2 inflammation, suggesting that opportunities to improve outcomes using a FeNO-guided approach were limited. A post-hoc analysis also found that algorithm recommendations were similar in both arms of the trial, suggesting good correlation between FeNO and symptoms in this study population.

A subgroup analysis of data from a primary care trial comparing FeNO- and symptom-guided strategies^17^ found that, in patients with a low FeNO at baseline, FeNO-guided management was associated with significantly lower ICS consumption and medication costs. However, FeNO-guided management was not associated with any significant changes in asthma control, acute exacerbations, or asthma-related quality of life.

## IMPACT OF FENO TESTING ON ASTHMA MANAGEMENT IN PRIMARY CARE

Although FeNO testing may initially lead to either increased or decreased medication costs when treatment adjustments are made, maintenance costs are likely to be reduced because of fewer follow-up visits and better control of asthma symptoms, resulting in an estimated saving of £114 million in 2023 from using FeNO to optimise asthma treatment.^2^ Additionally, avoiding incorrect asthma diagnoses through use of FeNO testing could save the NHS around £32 million.^2^

Between April 2021 and March 2023, all 15 Academic Health Sciences Networks (AHSNs) in England delivered a national programme to support introduction and integration of FeNO testing into primary care as part of the NHS England Accelerated Access Collaborative (AAC) Rapid Uptake Products (RUP) Programme.^18^ The programme included provision of equipment and consumables, two national training modules, and an implementation toolkit, and is estimated to have led to just over half of Primary Care Networks (PCNs) in England now having access to FeNO testing. The National FeNO programme impact report estimated that the programme supported around 58 000 new asthma diagnoses with around two-thirds of FeNO tests being used for diagnostic purposes and around one-third being used for monitoring.^18^

Pilot studies show that children, adults, and clinicians are receptive in principle to using FeNO in primary care and find it to be a feasible and acceptable test.^19^^,^^20^ Patients feel that including FeNO in their routine asthma review would help them understand their asthma better, facilitate more open discussions, and guide more tailored management of their asthma. Clinicians feel that FeNO could be a useful objective measure to help them provide more personalised management plans, educate patients about their asthma,^21^ and feel more confident about their clinical decisions.^20^

Clinicians have been shown to modify patients’ management plans in around one-third of cases after a FeNO result is made available to them. Around 90% of these changes relate to starting, stopping, or adjusting doses of ICS.^22^ FeNO-guided management decisions could potentially lead to a reduction in acute exacerbations in patients with high type-2 inflammation^10^ and reduced prescribing of asthma medications in patients with low type-2 inflammation without worsening clinical outcomes or quality of life.^17^

Identifying patients with high FeNO results despite high-dose ICS treatment may also lead to earlier referral of patients for consideration of biologic therapies. A FeNO-predominant type-2 inflammation profile is strongly predictive of a positive clinical response to treatments such as dupilumab and tezepelumab, which target airway rather than systemic inflammation.^8^

## IMPLEMENTATION OF FENO IN PRIMARY CARE: WHAT ARE THE NEXT STEPS?

### Recognising and addressing barriers to acceptance

Imbalances between institutional recommendations versus established professional practices and standards are well-recognised challenges when introducing new clinical practice guidelines and innovations, including FeNO testing.^23^ Potential barriers to acceptance include increasing complexity of diagnostic and management algorithms and care pathways, and concerns about clinic space requirements.^18^^,^^23^^,^^24^ Developing FeNO testing as an additional service may also require existing staff to take on more work, or new staff to be recruited and trained; this may be complicated without sufficient local funding or infrastructure.^24^

Additionally, differences between clinical practice guidelines can lead to confusion and non-adherence to recommendations. Neither British Thoracic Society/Scottish Intercollegiate Guidelines Network^12^ nor NICE^11^ guidelines recommend that FeNO should routinely be used to monitor patients with asthma. However, NICE recommends that FeNO testing should be considered to support asthma management in patients who have poorly controlled symptoms despite ICS treatment.

Non-adherence to algorithm recommendations was not found to have implications for numbers of exacerbations in children recruited from a combination of primary and secondary care settings.^16^ However, non-adherence to both step-up and step-down treatment recommendations generated by a composite type-2 biomarker-guided algorithm in patients with severe asthma led to worse clinical outcomes.^25^

Although there is evidence to suggest that FeNO can be used to help guide reductions in ICS treatment without increasing exacerbations in patients with mild-to-moderate asthma,^26^ the most common reason for non-adherence in primary care is not stepping down treatment when advised to do so.^27^

Future research should aim to explore these uncertainties in greater detail, understand how they can be addressed, and provide high-quality evidence to inform clear guidance to inform clinically and cost-effective models for using FeNO to guide asthma management in primary care.

### Building a sustainable future

The National FeNO programme impact report identified the need for strong clinical leadership and long-term infrastructure to provide the financial and logistical resources needed for FeNO testing to be incorporated into clinical pathways for asthma diagnosis and management in primary care.^18^ Asthma + Lung UK’s recent report on timely diagnosis of respiratory conditions^24^ particularly highlighted the need for Integrated Care Boards (ICBs) to develop and implement strategies to identify local population needs and make plans to ensure that sufficient workforce, equipment, training, and other resources are provided to meet these needs.

The 2017 NICE guideline^11^ was introduced at a time when most general practices did not possess FeNO analysers and were expected to purchase these from their own funds.^23^ Although the national FeNO programme considerably increased access to relevant equipment and consumables,^18^ the programme also identified major financial challenges to sustaining this access, including the absence of any ongoing funding or remuneration routes for FeNO testing.

Local initiatives designed to support and sustain FeNO testing services include provision of PCN-level respiratory champions, maximising staff resources by training lower-band members of staff to deliver testing and higher-band colleagues to interpret and report findings, setting up of primary care respiratory clinics that may run in a fixed location, rotate around practices, or review patients outside of usual working hours (when there is more space and flexibility), and introduction of referral pathways from primary care to local community hospital clinics that can provide support with diagnosis, treatment advice, and patient education.^24^ Such initiatives require collaborative working and operational pathways that are tailored according to local needs and availability of resources.

## CONCLUSIONS

FeNO testing can potentially provide a non-invasive, feasible way of delivering more personalised asthma management in the community using a ‘treatable traits’ approach. However, more research is needed to develop evidence-based, efficient, sustainable clinical pathways that maximise improvement in clinical decisions and outcomes compared with symptom- or guideline-based strategies. FeNO service delivery models that centralise staff and equipment should be optimised and tailored according to local resource availability and patients’ needs.
